# Placebo Devices as Effective Control Methods in Acupuncture Clinical Trials: A Systematic Review

**DOI:** 10.1371/journal.pone.0140825

**Published:** 2015-11-04

**Authors:** Claire Shuiqing Zhang, Hsiewe Ying Tan, George Shengxi Zhang, Anthony Lin Zhang, Charlie Changli Xue, Yi Min Xie

**Affiliations:** 1 Traditional and Complementary Medicine Research Program, RMIT Health Innovations Research Institute, School of Health Sciences, RMIT University, Bundoora, Victoria, Australia; 2 Centre for Innovative Structures and Materials, School of Civil, Environmental and Chemical Engineering, RMIT University, Melbourne, Victoria, Australia; Zhejiang Key Laborotory for Research in Assesment of Cognitive Impairments, CHINA

## Abstract

While the use of acupuncture has been recognised by the World Health Organisation, its efficacy for many of the common clinical conditions is still undergoing validation through randomised controlled trials (RCTs). A credible placebo control for such RCTs to enable meaningful evaluation of its efficacy is to be established. While several non-penetrating acupuncture placebo devices, namely the Streitberger, the Park and the Takakura Devices, have been developed and used in RCTs, their suitability as inert placebo controls needs to be rigorously determined. This article systematically reviews these devices as placebo interventions. Electronic searches were conducted on four English and two Chinese databases from their inceptions to July 2014; hand searches of relevant references were also conducted. RCTs, in English or Chinese language, comparing acupuncture with one of the aforementioned devices as the control intervention on human participants with any clinical condition and evaluating clinically related outcomes were included. Thirty-six studies were included for qualitative analysis while 14 were in the meta-analysis. The meta-analysis does not support the notion of either the Streitberger or the Park Device being inert control interventions while none of the studies involving the Takakura Device was included in the meta-analysis. Sixteen studies reported the occurrence of adverse events, with no significant difference between verum and placebo acupuncture. Author-reported blinding credibility showed that participant blinding was successful in most cases; however, when blinding index was calculated, only one study, which utilised the Park Device, seemed to have an ideal blinding scenario. Although the blinding index could not be calculated for the Takakura Device, it was the only device reported to enable practitioner blinding. There are limitations with each of the placebo devices and more rigorous studies are needed to further evaluate their effects and blinding credibility.

## Introduction

The use of acupuncture dates back as far as 1700BC from ancient China [1] and is currently an internationally used treatment option. In 2003, the World Health Organisation published a review and analysis of clinical controlled trials on acupuncture for a number of conditions [2] and recently there has been increasing attention in researching acupuncture needles [3–5]. Despite the frequent and wide utilisation, clinical trials have yielded conflicting results regarding the benefit of acupuncture [6]. There is a need for scientifically rigorous studies to evaluate the theoretical basis for acupuncture [7].

RCTs are considered the “gold standard” of evaluating the efficacy of an intervention. With regard to acupuncture RCTs, several types of control interventions have been used. These include 1). sham acupuncture, which involves skin penetration using the needles, either shallowly or on non-acupuncture points; 2). placebo acupuncture, which involves non-penetrating placebo acupuncture devices; 3). pseudo stimulation, such as transcutaneous electrical nerve stimulation (TENS) or laser acupuncture; and 4). other therapies or no treatment [8,9].

**Fig 1:**
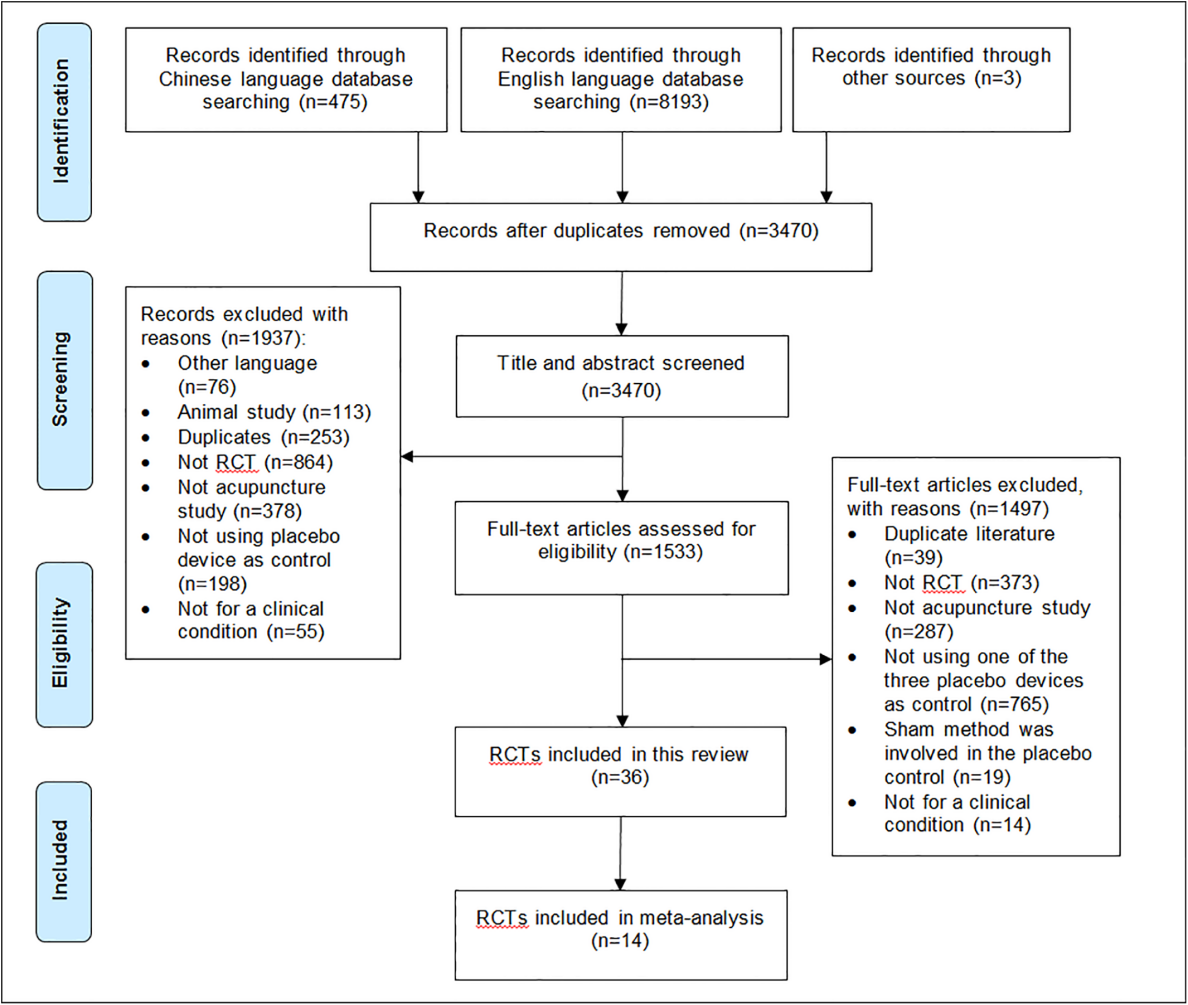
PRISMA flow diagram of study selection process.

Placebo-controlled studies needed to evaluate the efficacy of interventions. The ideal placebo control should be inert to enable the differentiation between the specific effects and non-specific effects of an intervention [10], yet indistinguishable from the real intervention to allow blinding of both practitioners and participants of RCTs as well as those involved in data gathering and analysis. However, when it comes to complex physical interventions such as acupuncture establishing an appropriate placebo-control intervention has been a major barrier as it is difficult to determine whether the so called “placebo” is fully inert. Sham and placebo needling have been two of the most commonly used forms of placebo-control in acupuncture studies. However, with the former, there is an on-going debate on its appropriateness as an inert control [11] as there have been studies suggesting the possibility of physiological responses elicited by skin penetration of the sham intervention [12]. To address the challenges of placebo control methods, researchers have also developed a number of non-penetrating placebo acupuncture devices. The first device, commonly known as the Streitberger Device, was introduced by Streitberger and Kleinhenz in 1998 [13]. The Streitberger Device uses a blunt-tipped needle with a shaft that telescopes into the copper handle of the needle, allowing the production of a pricking sensation when the needle touches the skin but without skin penetration. However, the Streitberger Device is said to be potentially unsuitable for certain areas of the body, does not allow for diversity in manual stimulation or needling direction and the sterilisation of needles may be compromised as the needle penetrates through the dressing plaster [14,15]. The Park Device was introduced shortly after and attempted to improve the design [16]. It includes a blunt-tip telescope needle within a standard guide tube, and a sheath (Park tube) with a flange connected at one end to maintain the sterilisation. The device is secured to the skin with double sided sticky tape. However, both the Streitberger Device and the Park Device do not allow for double-blinding. The Takakura Device was introduced in 2007 [17]. This device also utilises a blunt-tipped needle that touches but does not penetrate the skin; and a stopper is added to limit the depth of needle insertion to create a similar appearance to a penetrating needle. There is also added soft material stuffing in the guide tubes of the device to generate a similar feeling by practitioners during needling. The Takakura Device was the first acupuncture placebo device that was designed to enable the blinding of both practitioners and participants. Fig 1 presents these three devices.

While there are several other non-penetrating placebo acupuncture devices available [18–21], the above three have been the most widely used and validated in multiple RCTs. Currently, there has yet to be a comprehensive systematic review investigating whether these devices fulfil the requirements of being inert placebo controls in RCTs for different conditions. This article will fill the important knowledge gap by systematically reviewing RCTs of acupuncture which utilised one of these three placebo acupuncture devices, with the primary aim to evaluate their validity as an inert placebo intervention, from the points of view of minimising therapeutic effects and successful blinding. The results from this study may enable the comparison between the three placebo acupuncture devices, support further study into what makes a credible placebo acupuncture device and potentially lead to the development of a better form of acupuncture control intervention for future RCTs.

## Methods

### Search strategies

Electronic searches were carried out on four English databases (CINAHL, Cochrane Library, Embase, PubMed) and two Chinese databases (VIP Database for Chinese Technical Periodicals (CQVIP) and China National Knowledge Infrastructure (CNKI)) from their inceptions to July 2014. The search terms applied were in three groups: acupuncture, RCT, and placebo/sham. Search terms used in Pubmed search is provided as in supplementary file (S1 Table) as an example. Hand searches of references of relevant articles and publication lists of the key authors (Streitberger, Park, Takakura, and their co-authors in this field) were also conducted.

### Study selection criteria

Published RCTs, in English or Chinese language, comparing manual acupuncture with the Streitberger Device, the Park Device or the Takakura Device as the control intervention on human participants with any clinical condition and evaluating clinically related outcomes were included in this review. Since the purpose of this review is to evaluate the placebo devices, we did not place any limitation on the clinical conditions and their outcome measures. However, studies which modified the placebo acupuncture devices or did not apply the device as it was designed were excluded. Studies were also excluded if sham points were adopted in placebo acupuncture control groups in addition to placebo device. Finally, although electroacupuncture is one of the most frequently used methods in acupuncture clinical trials, the distinction or added-on effect from electric stimulation in electroacupuncture is unclear. Therefore, studies which applied techniques other than manual acupuncture, such as TENS, electroacupuncture or laser acupuncture were excluded to minimise confounding factors.

### Data Extraction and Risk of Bias Assessment

The publication year, disease or condition studied, participants’ demographic data, methodological characteristics, treatment protocol, clinically relevant outcomes, and evaluation of blinding, if available, were extracted from included studies onto an Excel spread sheet by two reviewers (HYT and CSZ) and crosschecked. For multiple armed studies, only data of the relevant interventions were extracted. Assessment of risk of bias was conducted using the Cochrane Collaboration’s tool for assessing risk of bias [22]. Any disagreement was resolved via discussion.

### Data Analysis

Cochrane Review Manager (RevMan 5.3) software was used for statistical analysis. Post-treatment outcome data were selected for data analysis. If sufficient data were present, pooled analysis was conducted, with subgroup analysis for each of the placebo acupuncture device. Dichotomous data were reported as risk ratio (RR) with 95% confidence intervals (CI), and continuous data were reported as mean difference (MD) with 95% confidence intervals (CI), where the outcomes were measured in the same way between trials. For trials reporting the same outcome measures but which used different methods, the standardised mean difference (SMD) was reported. The success of blinding was evaluated using the blinding index (BI) developed by Bang *et al*. where possible [23].

The PRISMA checklist is available as supplementary file (S1 Checklist).

## Results

**Fig 2:**
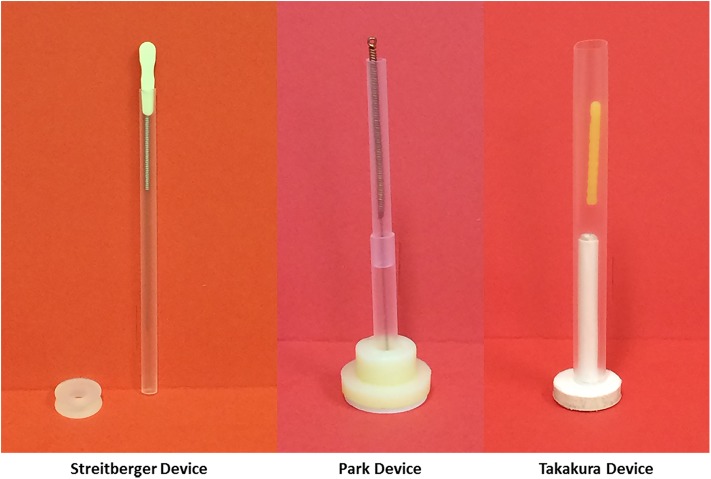
Placebo Acupuncture Devices.

The database searching yielded a total of 8,671 records. After duplicates were removed, the titles and abstracts of 3,470 articles were screened. 1,937 records were excluded for being duplicate studies, animal studies, non-RCTs, non-acupuncture studies, not employing a placebo acupuncture device as the control intervention, not involving a clinical condition or not published in English or Chinese. A total of 1533 full-text articles were retrieved for further evaluation, from which 36 were included in this review and 14 in the meta-analysis, respectively (Fig 2).

### Description of Included Studies

The 36 included studies were published between 1999 and 2013. Five studies were published in the Chinese language while the remaining 31 were published in English. Out of the 36 studies, 21 utilised the Streitberger Device [24–44], 13 utilised the Park Device [45–57] and two utilised the Takakura Device [58,59]. There were 21 studies on pain (musculoskeletal [25–27,29,30,32,39–41,46,48,52], headache [28,31], or induced pain/analgesia for a medical procedure [33–35,47,51,58,59]), four studies on in-vitro fertilisation (IVF) [24,42,53,54], two studies on obesity [56,57], two studies on chronic fatigue syndrome [43, 44], and one each on labour induction [49], tinnitus [50], chronic obstructive pulmonary disease (COPD) [55], Parkinson’s disease [46], emesis after chemotherapy [36], postmenopausal symptoms [37] and premature ejaculation [38]. The study sample sizes ranged from 10 to 635 participants, with seven crossover studies. The number of needles inserted varied from one to 16, with the treatment duration ranging from a one-off five minute treatment to a total of 600 minutes of treatment over a span on 12 weeks. The characteristics of included studies are summarised in Table 1.

**Table 1:** Characteristics of included studies.

Placebo Acupuncture Device	Condition	Author, Year	Total sample size /Dropout/ Analysed sample size	No. of acupuncture points	Treatment duration (per session)/No. of treatment sessions/Total treatment duration	Blinding credibility reported by the study	Primary outcome measures	Significant difference between effects of T & C
Streitberger Device	Pain (Carpel Tunnel Syndrome)	Yao, 2012	41/7/34	7	20min/6/6 weeks	NS	Carpel Tunnel Syndrome Assessment Questionnaire, CTSAQ (symptom and function scales)	NO
	Pain (1st metacarpophalangeal osteoarthritis pain)	Pariente, 2005	14/0/14 (Crossover)	1	24min/1/ NS	NS	Regional cerebral blood flow; Behavioural factors (Pain VAS; Holistic Health Questionnaire, HCAMQ; Needle Sensation Questionnaire, NSQ; Credibility Rating, CR)	NO
	Pain (Rotator cuff tendonitis)	Kleinhenz, 1999	52/7/52	12	20min/8/4 weeks	Tested—successful	Change in Constant-Murley score	YES (T>C)
	Pain (persistent arm pain due to repetitive use)	Goldman, 2008	123/5/123	7 to 10	20min/8/4 weeks	Tested—successful	Self-reported intensity of pain with movement on a 10-point numerical rating scale)	Yes (C>T)
	Pain (Chronic shoulder pain)	Lathia, 2009	20/3/20	8–16 (for traditional acu); 7 (for standard and sham acu)	20min/12/6 weeks	No	Shoulder Pain and Disability Index (SPADI)	YES (T>C)
	Pain (osteoarthritic pain)	White, 2011	147/8/113	average 6 points	20min/8/4 weeks	Tested—successful	Pain VAS	NO
	Pain (Knee osteoarthritis)	Chen, 2013	214/1? 213?	9	20min/12/6–12 weeks	Tested—successful	Change in Western Ontario and McMaster Universities Osteoarthritis Index (WOMAC); Secondary outcomes (Brief Pain Inventory, BPI; 36-item Short-Form Health Survey, SF-36; Patient Global Impression of Change; 6-minute walk test)	NO
	Pain (pelvic girdle pain in pregnant women)	Elden, 2008	115/7/115	13 to 15	30min/12/8 weeks	Tested—successful	Pain VAS	NO
	Pain (chronic/stable pain predominantly from a single joint (hip or knee) of known mechanical aetiology)	White, 2003	37/0/37 (crossover)	average 4 points	20min/4/2 weeks (washout 2 weeks)	Tested—successful	Questionnaire relating to needle sensation by Park; Secondary outcomes (Pain VAS; analgesia consumption; Nottingham Health Profile; Holistic Health Questionnaire; Credibility rating)	NO
	Pain (Pressure pain threshold in chronic tension-type headache)	Karst, 2000	39/0/39	max 15	30min/10/5 weeks	Tested—successful	Consumption of analgesics; Pain intensity VAS; site and duration of headache attacks; Clinical Global Impressions (CGI) scale; Nottingham Health Profile; Everyday-Life-Questionnaire; Freiburg Questionnaire of Coping with Illness; von Zerssen Depression Scale; Pressure pain thresholds	NO (for pain VAS and freq of headaches); YES (PPT significantly increased in verum acu)
	Pain (menstrual- related migraine)	Linde, 2004	31/3/28	12	30min/9/3 months	Tested—successful	Number of attacks per month; Secondary outcomes (days with migraine per month; mean headache intensity; amount of headache medication used)	NO
	Induced Pain (human pain models)	Rebhorn, 2012	50/0/50	8	1h20min/1/1 day	Tested—unsuccessful	Reduction in mean pain intensity during 3 minute cold-pressor test or mean pain intensity within 10 minutes after capsaicin injection	NO (Only yes for relief of capsaicin induced pain, but effects occurred mainly in a rating range that seems irrelevant to clinical pain)
	Induced Pain (Pressure pain detection threshold)	Schliessbach, 2011	45/0/45 (Crossover)	2	5min/1/1 day (10min washout)	NS	Pressure pain detection threshold (PPDT)	NO (between manual acu and manual NPSA)
	Induced Pain (Pressure pain detection threshold)	Schliessbach, 2012	45/0/45 (Crossover)	2	5min/1/1 day (10min washout)	NS	Pressure pain detection threshold (PPDT)	NO
	Antiemetic (in chemotherapy)	Streitberger, 2003	80/0/80	2	20min/2/2 days	Tested—successful	Number of patients who either had at least 1 episode of vomiting or required any rescue antiemetic drugs on the first day of high dose chemotherapy and the day after	NO
	IVF	Anderson, 2010	635/0/635	5 before ET; 4 after ET	30min/2/1 day	NS	ongoing pregnancy rate; live births	NO
	IVF	Zhang, 2003	140/0/140	4	25min/2/1 day	NS	Clinical pregnancy rate	YES (T>C)
	Postmenopausal symptoms and reproductive hormones	Sunay, 2011a	55/2/53	10	20min/10/5 weeks	NS	11 item Turkish version of the Menopause Rating Scale (MRS); Secondary (hormone levels)	YES (T>C)
	Premature ejaculation	Sunay, 2011b	60/0/60	10	20min/8/4 weeks	NS	Intravaginal ejaculation latency time (IELT), DSM-IV TR criteria, Premature Ejaculation Diagnostic Tool (PEDT)	YES (T>C)
	Chronic fatigue syndrome	Zhang, 2010	45/0/45	10	30min/20/4 weeks	NS	SF-20; Chalder Fatigue Scale	YES (T>C)
	Chronic fatigue syndrome	Zheng, 2011	80/3/77	14–16	30min/20/4 weeks	NS	SF-36, Health Utility	YES (T>C)
Park Device	Pain (Temporomandibular myofascial joint pain)	Smith, 2007	27/1/27	1	20min/6/3 weeks	Tested—successful	Patient functional perspective VAS; Pain intensity VAS; Pain distribution; Incisor opening and lateral movement measurement; Muscle tenderness; TMJ tenderness; Headaches; Deviation; TMJ Sounds	NS
	Pain (Non-specific low back pain)	Kennedy, 2008	48/3/45	8 to 13	30min/3–12/4–6 weeks	Tested—successful	Roland and Morris Disability Questionnaire, RMDQ); Pain VAS, Multidimensional patient-centred questionnaire	NO
	Pain (Pain intensity from a myofascial trigger point)	Chou, 2009	20/0/20	2	19min/1/1 day	NS	Numerical pain rating scale; Changes in endplate noise	YES (T>C)
	Pain (Analgesia during electromyography)	Smith, 2005	51/0/51	4	5min+-/1/1 day	Tested—successful	Pain VAS	NO
	Induced Pain (Thermal sensation and thermal pain thresholds)	Downs, 2005	18/0/18 (Crossover)	2	25min/1/3 weeks	Tested—12/18 answered correctly when asked what type of acupuncture received but may be correct guesses as it was not a statistically significant departure (P = 0.238)	Thermal sensation and thermal pain thresholds	NO
	Obesity	Tong, 2006	41/0/41	12	30min/20/40 days	NS	BMI; Hip circumference:height ratio	YES (T>C)
	Obesity	Tong, 2010	118/0/118	16	30min/12/5 weeks	NS	BMI; Area of adipose layer of abdomen; Area of adipose layer of thighs	YES (T>C)
	IVF	So, 2009	370/0/370	5 before ET; 4 after ET	25min/2/1 day	Tested—successful	Overall pregnancy rate (positive urinary pregnancy test)	Yes (C>T) for overall pregnancy rates; No for all other outcomes
	IVF	So, 2010	226/0/226	4	25min/1/1 day	Tested—successful	Overall pregnancy rate (positive urinary pregnancy test)	NO
	Labour induction	Modlock, 2010	125/0125	4	30min/1–2/1 day	Tested—successful	Labour/delivery	NO
	Tinnitus	Rogha, 2011	63/9/54	4 + accesory acupoints as needed	NS/10/3 weeks	NS	Tinnitus severity index; Tinnitus loudness questionnaire; Hospital anxiety and depression scale, HADS	YES (T>C)
	COPD	Suzuki, 2012	68/6/62	11	50min/12/12 weeks	NS	Breathlessness (10-point Borg category ratio scale)	YES (T>C)
	Parkinsons	Chae, 2009	10/0/10 (crossover)	1	1min/1/ NS	Tested—successful	fMRI scans; credibility data (Bang's index)	NO
Takakura Device	Pain (analgesia after third molar surgery)	Vase, 2013	101/0/101	5	30min/1/1 day	Tested—successful	Pain measures (Perceived pain intensity and pain unpleasantness M-VAS); Expectancy measures (expected pain intensity and pain unpleasantness M-VAS); perception of treatment allocation	YES (T>C)
	Induced pain	Takakura, 2009	56/0/56 (Crossover)	1	20min/1/1 day (24h washout)	Tested—successful	Pain elicited when electrical stimulation was applied (numeric rating scale 0–15); Secondary outcome (pain from skin penetration and the deqi associated with needle application, VAS)	NO

### Risk of Bias Assessment

**Fig 3:**
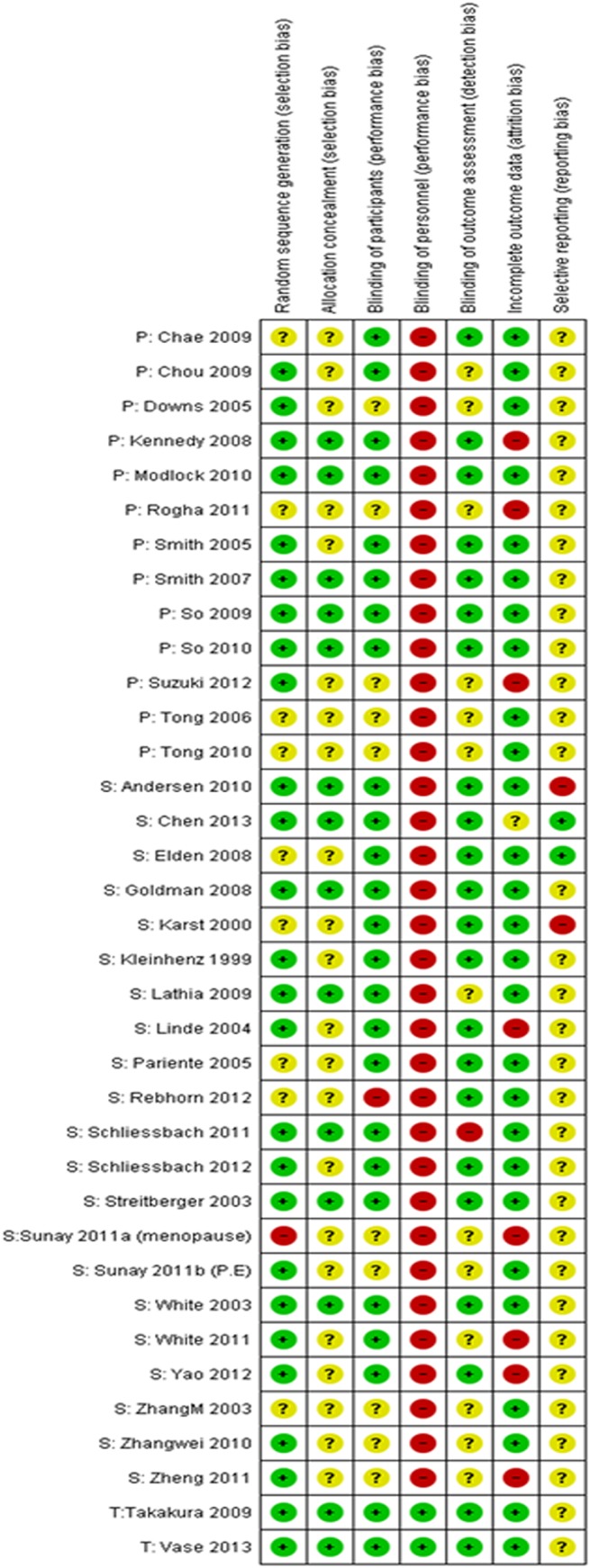
Risk of bias assessment of included studies. Note: studies were categorised according to the type of device (P = Park device, S = Streitberger device, T = Takakura device).

The overall risk of bias assessment is summarised in Table 2. In total, 118 “Low risk” assessment, 87 “Unclear risk” and 47 “High risk” were given to all 36 RCTs for seven domains. With regard to the blinding issue as the particular interest of this research, 69.4% (n = 25) and 61.1% (n = 22) of studies were judged with low risk for participant blinding and outcome assessment blinding, respectively. However, only 5.6% (n = 2) of studies which used the Takakura Device were low risk for blinding of personnel (acupuncturist), while the rest were given judgement of high risk. This highlights that practitioner blinding is a major issue that needs to be addressed to enable double-blinded acupuncture clinical studies. When the risk of bias assessment was analysed according to the different placebo device controls (Fig 3), studies using the Takakura Device were judged with low risk for all domains, except for selective reporting which was judged with unclear risk. Studies involving the Streitberger and Park Devices had similar distribution of high, low and unclear risks of bias. However, it should be noted that there were only two studies using the Takakura Device. Nevertheless, the biggest contrast shown in this comparison is the ability of the Takakura Device to enable personnel (acupuncturist) blinding.

**Table 2:** Risk of bias assessment of included studies.

Placebo device used by the study	Risk or bias assessment	Random sequence allocation	Allocation concealment	Blinding of personnel	Blinding of participants	Blinding of outcome assessors	Incomplete outcome data	Selective reporting
Streitberger Device	Low risk	15 (71.4%)	7 (33.3%)	15 (71.4%)	0 (0%)	13 (61.9%)	15 (71.4%)	2 (9.5%)
	Unclear risk	5 (23.8%)	14 (66.7%)	5 (23.8%)	0 (0%)	7 (33.3%)	1 (4.8%)	17 (81%)
	High risk	1 (4.8%)	0 (0%)	1 (4.8%)	21 (100%)	1 (4.8%)	5 (23.8%)	2 (9.5%)
Park Device	Low risk	9 (69.2%)	5 (38.5%)	8 (61.5%)	0 (0%)	7 (53.8%)	10 (76.9%)	0 (0%)
	Unclear risk	4 (30.8%)	8 (61.5%)	5 (38.5%)	0 (0%)	6 (46.2%)	0 (0%)	13 (100%)
	High risk	0 (0%)	0 (0%)	0 (0%)	13 (100%)	0 (0%)	3 (23.1%)	0 (0%)
Takakura Device	Low risk	2 (100%)	2 (100%)	2 (100%)	2 (100%)	2 (100%)	2 (100%)	0 (0%)
	Unclear risk	0 (0%)	0 (0%)	0 (0%)	0 (0%)	0 (0%)	0 (0%)	2 (100%)
	High risk	0 (0%)	0 (0%)	0 (0%)	0 (0%)	0 (0%)	0 (0%)	0 (0%)
**All studies**	**Low risk**	**26 (72.2%)**	**14 (38.9%)**	**25 (69.4%)**	**2 (5.6%)**	**22 (61.1%)**	**27 (75%)**	**2 (5.6%)**
	**Unclear risk**	**9 (25%)**	**22 (61.1%)**	**10 (27.8%)**	**0 (0%)**	**13 (36.1%)**	**1 (2.8%)**	**32 (88.9%)**
	**High risk**	**1 (2.8%)**	**0 (0%)**	**1 (2.8%)**	**34 (94.4%)**	**1 (2.8%)**	**8 (22.2%)**	**2 (5.6%)**

Note: results are reported as number (and %) of studies received the assessment

### Treatment Effects

Author-reported differences in therapeutic effects by primary outcome measures are summarised in Table 3. Among all studies, 20 studies (55.6%) reported no significant differences between verum acupuncture and the placebo devices, 13 studies (36.1%) reported verum acupuncture being more effective than placebo, and two studies (5.6%) were in reverse. A consistent trend was found when grouping studies according to the type of placebo devices (Table 3).

**Table 3:** Reported difference in effects between verum acupuncture and placebo devices.

Placebo Device	Significant difference in effects (Verum > Placebo)	Significant difference in effects (Placebo > Verum)	No significant difference in effects when compared to verum acupuncture	Not stated/Unable to evaluate	Total number of studies
Streitberger	7 (33.3%)	1 (4.8%)	13 (61.9%)	0 (0%)	21
Park	5 (38.5%)	1 (7.7%)	6 (46.2%)	1 (7.7%)	13
Takakura	1 (50%)	0 (0%)	1 (50%)	0 (0%)	2
**Total**	**13 (36.1%)**	**2 (5.6%)**	**20 (55.6%)**	**1 (2.8%)**	**36**

Meta-analysis was performed to multiple studies which were of same clinical conditions and reported same outcome measures (Table 4).

**Table 4:** Treatment effects of meta-analysis results.

Clinical condition (Outcome Measures)	Subgroups by Placebo Device	Number of studies	Treatment effect meta-analysis results
Pain–Musculoskeletal (Pain intensity using a VAS scale)	Streitberger	1 (24)	MD: -0.80, 95% CI [-1.54, -0.06] Verum acupuncture < placebo
	Park Device	2 (43, 45)	MD: 3.79, 95% CI [2.91, 4.67]. I^2^ = 0% Verum acupuncture > placebo
	**All studies**	**3 (24, 43, 45)**	**MD: 1.46, 95% CI [-2.92, 5.84]. I** ^**2**^ **= 0%, I** ^**2**^ **= 97%**
Pain–Headache (Pain intensity using a VAS scale)	Streitberger	2 (25, 28)	MD: -0.57, 95% CI [-1.11, -0.04]. I^2^ = 40% Verum acupuncture < placebo
Pain–Induced (Pressure pain threshold)	Streitberger	3 (30–32)	SMD: -0.06, 95% CI [-0.32, 0.20]. I^2^ = 0% Verum acupuncture = placebo
Pain–Induced (Pain intensity using a 10-point Numeric Rating Scale)	Streitberger	2 (31, 32)	MD: -1.41, 95% CI [-1.82, -1.00], I^2^ = 0% Verum acupuncture < placebo
Obesity (Body mass index)	Park Device	2 (53, 54)	MD: 2.50, 95% CI [1.57, 3.42], I^2^ = 48% Verum acupuncture > placebo
In-vitro Fertilisation (Clinical Pregnancy rates)	Streitberger Device	2 (21, 39)	RR: 0.85, 95% CI [0.49, 1.48], I^2^ = 79% Verum acupuncture = placebo
	Park Device	2 (50, 51)	RR: 1.25, 95% CI [1.03, 1.51], I^2^ = 0% Verum acupuncture < placebo
	**All studies**	**4 (21, 39, 50, 51)**	**RR: 1.07, 95% CI [0.84, 1.35], I** ^**2**^ **= 62% Verum acupuncture = placebo**
In-vitro Fertilisation (Overall pregnancy rates)	Park Device	2 (50, 51)	RR: 1.24, 95% CI [1.04, 1.47], I^2^ = 0% Verum acupuncture < placebo
In-vitro Fertilisation (Ongoing pregnancy rates)	Streitberger Device	1 (21)	RR: 1.17, 95% CI [0.92, 1.50] Verum acupuncture = placebo
	Park Device	2 (50, 51)	RR: 1.28, 95% CI [1.03, 1.59], I^2^ = 0% Verum acupuncture < placebo
	**All studies**	**3 (21, 50, 51)**	**RR: 1.23, 95% CI [1.04, 1.45], I** ^**2**^ **= 0% Verum acupuncture < placebo**
In-vitro Fertilisation (Live birth rates)	Streitberger Device	1 (21)	RR: 1.19, 95% CI [0.92, 1.53] Verum acupuncture = placebo
	Park Device	2 (50, 51)	RR: 1.26, 95% CI [1.00, 1.59], I^2^ = 0% Verum acupuncture < placebo
	**All studies**	**3 (21, 50, 51)**	**RR: 1.23, 95% CI [1.03, 1.45], I** ^**2**^ **= 0% Verum acupuncture < placebo**

#### Pain–musculoskeletal

There were 12 studies on musculoskeletal pain, three of which provided sufficient data of pain intensity measured using a 100mm visual analogue scale (VAS) or an instrument using a 10-point numerical rating scale (NRS). The VAS rating was converted to centimetres so that all ratings would be out of 10. Out of the three studies included in the meta-analysis, one study utilised the Streitberger Device as the control intervention [27] while two studies utilised the Park Device [46,48]. The overall meta-analysis showed that there were no significant differences between the verum acupuncture and the placebo devices on pain intensity VAS (MD: 1.46, 95% CI [-2.92, 5.84]). Subgroup analysis showed that the verum acupuncture significantly improved pain intensity VAS compared to the Park Device (MD: 3.79, 95% CI [2.91, 4.67]), however the Streitberger Device performed significantly better than the verum acupuncture (MD: -0.80, 95% CI [-1.54, -0.06]).

#### Pain–headache

The two included studies on headache evaluated pain intensity using a 10cm VAS [28,31]. Both studies utilised the Streitberger Device as the control intervention. Meta-analysis showed significant difference, favouring the Streitberger Device (MD: -0.57, 95% CI [-1.11, -0.04], I^2^ = 40%).

#### Pain–induced

Five studies were on induced pain and two on analgesia for a medical procedure. Among these seven studies, three studies which used the Streitberger Device evaluated pressure pain threshold (PPT) [33–35]. Standard mean difference (SMD) was calculated because one of the studies reported PPT as log data [33]. The meta-analysis showed that there was no significant difference between verum acupuncture and the Streitberger Device in increasing PPT (SMD: -0.06, 95% CI [-0.32, 0.20], I^2^ = 0%).

Two of the studies on induced pain also evaluated pain intensity using a 10-point NRS [34,35]. Both these studies were by the same authors and utilised the Streitberger Device as the study control intervention. Meta-analysis showed significant difference, favouring the Streitberger Device (MD: -1.41, 95% CI [-1.82, -1.00], I^2^ = 0%).

#### Obesity

The two studies on acupuncture for treating obesity evaluated body mass index (BMI) as one of the outcome measures [56,57]. Both studies utilised the Park Device as the control intervention. The meta-analysis showed significant difference, favouring verum acupuncture (MD: 2.50, 95% CI [1.57, 3.42], I^2^ = 48%).

#### In-vitro fertilization

There were four studies on acupuncture for IVF–two utilised the Streitberger Device [24,42] and two applied the Park Device [53,54].

Of the four studies, the two studies which employed the Park Device as the study control were by the same authors and evaluated overall pregnancy rates. Meta-analysis showed that the Park Device was significantly more effective than verum acupuncture (RR: 1.24, 95% CI [1.04, 1.47], I^2^ = 0%). All four studies evaluated clinical pregnancy rates, with the overall meta-analysis showing no significant difference between verum acupuncture and the placebo devices (RR: 1.07, 95% CI [0.84, 1.35], I^2^ = 62%). Three of the studies evaluated ongoing pregnancy rates and live birth rates as well [24,53,54]. Meta-analysis showed that there was similar significant difference in both these outcomes (RR: 1.23, 95% CI [1.04, 1.45], I^2^ = 0%; and RR: 1.23, 95% CI [1.03, 1.45], I^2^ = 0%), favouring the placebo devices. However, when looking at the subgroup analysis for clinical pregnancy rates, ongoing pregnancy rates and live birth rates, the Park device also showed significantly better effects than verum acupuncture, but the Streitberger device was not different to verum acupuncture. It should be noted that there was only one study using the Streitberger Device [24] that was included in the meta-analysis for ongoing pregnancy rates and live birth rates.

### Adverse events

Out of the 36 included studies, 20 did not mention the evaluation of occurrence of adverse events, while seven studies noted that no adverse events were observed or recorded. Nine studies (three using the Park Device [53–55], six using the Streitberger Device [25–27,29,31,39]) noted minor, mild or moderate side effects, with most reporting no significant difference between groups. One study noted significantly higher incidence of adverse events in the verum acupuncture group compared to the placebo (Streitberger) device acupuncture group [25]. However, the authors noted that acupuncture was given immediately after exercise-based physical therapy and it is therefore impossible to determine the exact cause of the side effects. One study noted no significant difference between the adverse events that occurred during the run-in and treatment period; however, there was significant difference (P = 0.004) in “new side effects attributable to acupuncture only in the treatment period” [27]. Another study also noted no significant difference in adverse effects, except for a significantly higher sensation of *Deqi* in the verum acupuncture group [26]. The total number of adverse events reported by studies is summarised in Table 5. Overall there were more adverse events occurred in the verum acupuncture group than in placebo acupuncture group (457 vs 331). The types of adverse events reported include nausea, dizziness/vertigo, fainting, tiredness/fatigue, drowsiness, headache, chest pain, puncture site itching, pain, bleeding/bruising, agitation, increased muscle tension/soreness, loss of strength in legs, tearful, inflammation/redness/infection and facial/cervical blush. The most commonly reported adverse event was acupuncture site itching among participants who received verum acupuncture and drowsiness among those who received treatment with the placebo devices. Nevertheless, none of the studies provided further elaboration regarding the occurrence in adverse events or whether it affected participant or practitioner blinding.

**Table 5:** Summary of adverse events reported by RCTs.

Studies grouped by Placebo Device	Total number of events (n =)	Events in verum acupuncture group (n =)	Events in placebo acupuncture group (n =)
Streitberger (10 studies)	338	208	130
Park (4 studies)	450	249	201
Takakura (1 study)	0	0	0
**All studies (15 studies)**	**788**	**457**	**331**

### Blinding credibility

Out of the 36 studies, 15 did not mention credibility blinding [24,32,34,35,37,38,41–44,46,50,55–57] and one study mentioned that blinding credibility was not evaluated [30]. Twenty studies conducted credibility testing, 19 of which reported successful blinding [25–29,31,36,37,40,46–49,51–54,58,59] and one of which had unsuccessful blinding [33]; however, the authors emphasized that incomplete blinding did not affect the results. Table 6 summarises the number of author-reported blinding credibility testing for each of the placebo devices.

**Table 6:** Author reported blinding credibility testing.

Placebo device used in the study	Blinding credibility tested–successful (n)	Blinding credibility tested–unsuccessful (n)	Blinding credibility not tested (n)	No mention of blinding credibility testing (n)	Total (n)
Streitberger	9	1	1	10	21
Park	8	0	0	5	13
Takakura	2	0	0	0	2
**All studies**	**19**	**1**	**1**	**15**	**36**

Only two studies which utilised the Streitberger Device [26,42] and five studies which employed the Park Device [45,48,49,53,57] had sufficient data to enable the calculation of the BI (Table 7). Using the rule of thumb based on a 0.2 BI cut off point and the “classification rules of nine blinding scenarios” [60,61], the BI calculation showed that out of the seven studies, only one study which utilised the Park Device [49] could possibly have had ideal blinding and clinical effectiveness interpretations. “Unblinded participants” in the verum acupuncture group (BI>0.2) and “opposite guesses of participants” in the placebo group (BI<-0.2) was found in the other six individual studies [26,32,45,48,53,54], as well as the pooled BI results of studies used Streitberger Device [26,32] and that of studies used Park Device [45,48,49,53,54].

**Table 7:** Blinding index.

Placebo device	Author, year	Guess real in AC group	Unsure in AC group	Guess placebo in AC group	Guess real in CT group	Unsure in CT group	Guess placebo in CT group	VBI	SBI	VBI vs SBI	Clinical effectiveness interpretations
**Streitberger**	Pariente 2005	14	0	0	11	0	3	1.00	-0.57	Unblinded vs Opposite guess	Possible that patients tend to have wishful thinking, weak treatment and strong placebo effect, or any treatment administered is perceived as real treatment
	Elden 2008	35	18	1	35	15	2	0.63	-0.63	Unblinded vs Opposite guess	
	**All studies**	**49**	**18**	**1**	**46**	**15**	**5**	**0.71**	**-0.62**	Unblinded vs Opposite guess	
**Park**	So 2009	111	59	15	95	61	29	0.52	-0.36	Unblinded vs Opposite guess	Possible that patients tend to have wishful thinking, weak treatment and strong placebo effect, or any treatment administered is perceived as real treatment
	Modlock 2010	8	26	5	5	28	7	0.08	0.05	Random guess vs Random guess	Ideal scenario
	So 2010	79	28	6	55	32	26	0.65	-0.26	Unblinded vs Opposite guess	Possible that patients tend to have wishful thinking, weak treatment and strong placebo effect, or any treatment administered is perceived as real treatment
	Chae 2009	9	0	1	7	2	1	0.80	-0.60	Unblinded vs Opposite guess	
	Kennedy 2008	23	0	0	22	0	0	1.00	-1.00	Unblinded vs Opposite guess	
	**All studies**	**230**	**113**	**27**	**184**	**123**	**63**	**0.55**	**-0.33**	Unblinded vs Opposite guess	

Note: AC: acupuncture; CT: control; BI: blinding indext; VBI: blinding index of real acupuncture group; SBI: blinding index of sham acupuncture group. The interpretation of BI results is based on the 9 blinding scenarios introduced by Freed et al 2014 [61]

## Discussion

The three most frequently used placebo devices have been used in RCTs for a variety of conditions, with pain being the most common condition, followed by IVF. The number of studies somewhat reflects the length of time that the placebo device has been made available, with the majority of the studies using the Streitberger Device and the least studies using the Takakura Device.

The ideal acupuncture placebo device should be fully inert and support participant blinding to reduce placebo effects. In terms of the efficacy, a recent meta-analysis of individual patient data of acupuncture RCTs for pain found that, there were differences in effect sizes among trials with different control conditions. This implies that trials used non-penetrating needle control had overall larger effect size compared to those using penetrating needle sham control [62]. However, this review only evaluated RCTs of pain conditions. The meta-analyses of our review showed that there were no significant differences between the therapeutic effects by the Streitberger Device when compared to verum acupuncture. With regard to the Park Device, the meta-analyses showed that verum acupuncture was significantly more effective, except in the cases of IVF, where the Park Device were significantly more effective. The overall analysis does not support the notion of these devices being an inert control intervention, although it may be debated that the Park Device shows more promise compared to the Streitberger Device. However, most studies noted that the placebo devices may not be a completely inert intervention Nevertheless, the number studies which were included in the analyses was small and these studies were of poor quality as evaluated by the risk of bias assessment and should be interpreted with caution. Furthermore, if a no treatment (waiting list) group was included in these RCTs, the difference between the placebo group and the no treatment group may further assist in evaluating the validity of placebo intervention. Unfortunately, only one RCT [42] employed a waiting list group as the third arm. Further research should take this point into consideration.

Out of the 16 studies that reported adverse events, only one study noted significantly more adverse events by verum acupuncture when compared to the Streitberger Device [25]. All other reported adverse events were deemed minor, with no significant difference between verum acupuncture and any of the placebo devices. Generally, verum acupuncture seemed to have more incidences of most types of adverse events reported. However, it is interesting to note that despite being non-penetrating devices, there were still adverse events reported among participants in the placebo groups. It should be noted there were no reports of pain as an adverse event caused by the Park Device, while it was fairly common with the Streitberger Device. This may be a difference in reporting by authors, as there were reports of ‘puncture site itching’ by the Park Device instead.

When blinding credibility was reported, most authors claimed successful blinding. However, several studies reported blinding credibility vaguely, stating that no participants were able to distinguish between verum and placebo acupuncture instead of reporting the exact number of participants guessing the intervention correctly or incorrectly. In our study, the BI calculated for the seven studies did not strongly support the notion of successful blinding. Only one study which utilised the Park Device [49] could possibly have had ideal blinding scenario. However, the pooled BI results of Streitberger Device and Park Device were not indicating an ideal blinding scenario. While BI could not be calculated for the Takakura Device, the authors of the two studies reported successful participant blinding and it was the only device which was able to support practitioner blinding as well. Recently, Moroz *et al*. used BI to evaluate the effectiveness of blinding of 54 acupuncture RCTs [63]. It was found that the studies (n = 22) using three non-penetrating needles as placebo control (Streitberger, Park, and Takakura devices) achieved effective blinding of participants. However, this study did not perform subgroup analysis to investigate the difference among these three devices [63]. In addition, after the completion of our research, a systematic review assessing non-penetrating placebo needles was published [64], which concluded that non-penetrating placebo needles achieved effective blinding. Unfortunately the number of included studies was very small (n = 5), and the authors did not differentiate three types placebo devices in their analysis.

Originally, BI was demonstrated with pharmacological studies [23], and recently it has been used in acupuncture studies to assess the blinding credibility [65–67]. Since BI is directly interpreted as the percentage of un-blinding beyond chance, it can capture different behaviours in different arms. Particularly, BI may reveal the ‘wishful thinking’ or ‘lack of idea about control treatment’ scenario in which patients believe they are on active treatment. These scenarios are common in acupuncture studies [60]. In fact, the interpretation of BI can be subjective because this may represent complete blinding or complete un-blinding in opposite directions. The cut-off points, whether it is 0.2 or 0.3 is also somehow subjective. Further research using BI should carefully address such complexity.

When comparing the design of the three placebo acupuncture devices, the Streitberger has been the most widely used and validated. Despite being shown to be successful in participant-blinding, it does not solve the problem of practitioner- or double-blinding. Furthermore, concerns were raised regarding the difficulty in applying the device on acupuncture points in certain areas such as the fingers, toes and scalp [14]. Also, it does not allow for a variation in needle manipulation or direction of insertion. Furthermore, it was stated that the needle sterilisation may be compromised as the needle penetrates through the dressing plaster [15]. In one study, practitioners complained about the limitation of choosing acupuncture points and the need to apply acupuncture using the ring and dressing plaster so that real and placebo acupuncture appeared the same [27]. Another study noted that the use of the ring and plaster may increase discomfort in participants and limit the type of needling techniques [41].

The Park Device does not support double-blinding either and shares the limitation of the Streitberger Device where there is difficulty in applying the device at points located on the toes [49], fingers and scalp. However, the added oversized guide tube and silicon flange in the Park Device prevent the compromise of needle sterilization and is said to allow the practitioner to perform manipulation as necessary [16].

The Takakura Device is reported to be applicable at all acupuncture points, including those on the toes, fingers and scalp and the practitioner is able to alter direction of needle insertion by moving the lower end of the guide tube [68]. Being the newest among the three devices, the Takakura Device is mostly praised for being the first placebo acupuncture device to enable practitioner blinding. This is because of the soft material stuffing that Takakura and colleagues added into the guide tube of the device, to ensure that the practitioner experiences the same sensations when inserting verum acupuncture needles or the blunt-tipped non-penetrating needle. However, in order to ensure a uniform appearance and insertion depth, the Takakura Device is made with a stopper to limit the depth of needle insertion. While a variety of needle lengths differentiated by colour coded handles can be easily produced, it may increase the costs of production. Furthermore, researchers using the Takakura Device would not have the choice of needles, as they would when using the Streitberger or Park Devices. Upon examination of the Takakura Device, we have noted that the soft stuffing used is quite firm, thereby causing the practitioner to feel the same amount of tension when needling with a real needle or with the placebo device. However, this tension is stronger than what a practitioner would normally experience with verum acupuncture. Both the stuffing and stopper in the Takakura Device also limit the ability for needle manipulation and the ability of the practitioner to feel *Deqi* sensation during needling.

In all cases of placebo acupuncture devices, unblinding could occur if there was any bleeding cause by verum acupuncture. However, in this review, it was noted that there were several cases of bleeding or bruising by the Streitberger Device as well [25,26,31,39]. Another concern is with regard to the stimulation or physiological effects from the touching of the skin by the blunt-tipped needles. In efforts to overcome this, Takakura *et al*. designed a modified “no touch” version of the Takakura Device, whereby the “the tip of the placebo needle does not penetrate through the stuffing to come in contact with the skin [69]. However, a validation study showed that this device did not support participant blinding and was, therefore, not suitable for double-blind testing of acupuncture effects [69,70].

With the improvements in the Takakura Device, it appears that practitioner-blinding is also made possible. However, traditional acupuncture (notwithstanding variation in practice based on country or school of thought) requires the practitioner to be able to insert the needle at various locations with different angles, depth and manipulation. Minimising the size of the flange may reduce concerns regarding the discomfort felt by participants and altering the flange to include a pivot device may overcome the issue of needling at various locations and angles. In addition, the stopper used in the Takakura Device may be omitted and the current stuffing could be replaced with a softer material to enable better control of the depth of insertion and manipulation of needles. An alternative would be to incorporate the telescoping blunt-tipped needle with added stuffing in the telescoping handle to the Takakura Device so that the practitioner may still experience the same sensation as the verum acupuncture.

From this review, aside from highlighting the need for placebo controls to be inert and support blinding, it should be noted that the placebo controls should also enable the real intervention to be performed as per normal and for the placebo to mimic its appearance and experience felt by practitioners and participants. Furthermore, with acupuncture studies, the expectation of creating an inert placebo control is related to the assumption that acupuncture is indeed an efficacious treatment.

Previous studies on acupuncture mechanism suggest that acupuncture effects are due to physiological response and nervous activation by needle insertion [71]. Therefore, non-penetrating placebo devices were said to be the potential solution to this issue. However, Dorsher argues that true “sham” needles should produce a sensation which mimics that of verum acupuncture [72]. He further claims that these devices are likely to produce no significant difference in outcomes when compared to verum acupuncture, as seen with some of the meta-analyses in this study. Although it has been acknowledged that these non-penetrating acupuncture placebo devices are not fully inert, they seem to have been fairly successful in participant-blinding and are considered the current best available form of acupuncture placebo control.

Our research found that there is yet insufficient evidence to identify “the best” placebo device from among the three devices which have been evaluated in this review. As the current state of evidence of the efficacy of acupuncture remains unclear, it is still debatable whether it is possible, or even necessary, to achieve a placebo control for the intervention; or whether it would be more beneficial to evaluate the effectiveness of acupuncture in comparison to other therapies instead [73].

It should be noted that other confounding factors, e.g. participant expectation/experience, and practitioner-participant interaction, may affect therapeutic effect and blinding [74]. In our review, the majority of included studies failed to clearly report on whether these issues were considered and what precautions were taken. Future RCTs should report more details on how much information was given to participants regarding the interventions, whether or not participants were acupuncture naive, and how practitioner-participant interactions were limited/encouraged.

## Conclusions

Based on the meta-analyses, neither the Streitberger Device nor the Park Device seemed to be an adequate inert control for acupuncture RCTs, while none of the studies which utilised the Takakura Device were included in the meta-analyses to allow for comparison. Author-reported blinding credibility apparently showed that all three placebo devices were mostly successful in participant blinding; however, when comparing the blinding index, only one study, which utilised the Park Device, was noted to have an ideal blinding scenario. To date, the Takakura Device is the only device that seemed to enable practitioner blinding and may therefore seem to have more promise as a suitable placebo control. With these in mind, more rigorous studies are needed to further evaluate its effects when compared to verum acupuncture and its blinding credibility. There are limitations with each of the devices and more research is needed to inform the future development of an improved placebo device for future acupuncture RCTs.

## Supporting Information

S1 Checklist PRISMA Checklist. (DOC)

S1 Table Search strategy. (DOCX)

## References

[1] Lao L. Acupuncture techniques and devices. Journal of Alternative and Complementary Medicine. 1996;2 (1):23–5. . 9395637 10.1089/acm.1996.2.23

[2] WHO. Acupuncture: Review and Analysis of Reports on Controlled Clinical Trials. Geneva: WHO Publications; 2003.

[3] Xie YM, Xu S, Zhang CS, Xue CC. Examination of surface conditions and other physical properties of commonly used stainless steel acupuncture needles. Acupuncture in Medicine. 2014 4;32(2):146–54. 10.1136/acupmed-2013-010472 24522003 PMC3995252

[4] Zhang CS, Pannirselvan M, Xue CC, Xie YM. Relationship between buckling of acupuncture needles and the handle type. Acupuncture in Medicine. 2014 10;32(5):400–5. 10.1136/acupmed-2014-010586 25134426

[5] Zhang CS, Zhang AL, Xue CC, Xie YM. New approach to preventing long acupuncture needles from buckling and contamination during insertion. Acupuncture in Medicine. 2014 12;32(6):520–2. 10.1136/acupmed-2014-010671 25315115

[6] Zhu D, Gao Y, Chang J, Kong J. Placebo acupuncture devices: Considerations for acupuncture research. Evid-Based Complement Altern Med. 2013;2013. 10.1155/2013/628907 PMC3690239 23840261

[7] Moffet HH. Traditional acupuncture theories yield null outcomes: a systematic review of clinical trials. J Clin Epidemiol. 2008 8;61(8):741–7. 10.1016/j.jclinepi.2008.02.013 Epub 2008 Jun 6. 18538996

[8] Vickers AJ. Placebo controls in randomized trials of acupuncture. Eval Health Prof. 2002;25(4):421–35. 10.1177/0163278702238055 12449085

[9] Birch S. Overview of models used in controlled acupuncture studies and thoughts about questions answerable by each. Clin Acupunct Oriental Med. 2003;3(4):207–17. 10.1016/S1461-1449(02)00044-0

[10] Dincer F, Linde K. Sham interventions in randomized clinical trials of acupuncture—A review. Complement Ther Med. 2003;11(4):235–42. 10.1016/S0965-2299(03)00124-9 15022656

[11] Moffet HH. Sham acupuncture may be as efficacious as true acupuncture: A systematic review of clinical trials. Journal of Alternative and Complementary Medicine. 2009;15(3):213–6. 10.1089/acm.2008.0356 19250001

[12] Näslund J, Lundeberg T, Lund I, Sing A. Is placebo acupuncture what it is intended to be? Evid-Based Complement Altern Med. 2011;2011. 10.1093/ecam/nep049 PMC3139519 19525330

[13] Streitberger K, Kleinhenz J. Introducing a placebo needle into acupuncture research. Lancet. 1998;352(9125):364–5. Epub 1998/08/26. 10.1016/s0140-6736(97)10471-8 . 9717924

[14] Kaptchuk TJ. Placebo needle for acupuncture. Lancet. 1998;352(9132):992 Epub 1998/09/30. 10.1016/s0140-6736(05)61551-6 . 9752848

[15] Park J. Sham needle control needs careful approach. Pain. 2004;109 (1–2):195–6. . 15082143 10.1016/j.pain.2004.02.010

[16] Park J, White A, Lee H, Ernst E. Development of a new sham needle. Acupuncture in Medicine. 1999;17(2):110–2.

[17] Takakura N, Yajima H. A double-blind placebo needle for acupuncture research. BMC complementary and alternative medicine. 2007;7:31 10.1186/1472-6882-7-31 . 17925042 PMC2176062

[18] Goddard G, Shen Y, Steele B, Springer N. A controlled trial of placebo versus real acupuncture. Journal of Pain. 2005;6 (4):237–42. 10.1016/j.jpain.2004.12.009 . 15820911

[19] Kim S. Creating an instrument for a successful double-blind acupuncture placebo. JAMS Journal of Acupuncture and Meridian Studies. 2008;1(1):36–41. 10.1016/s2005-2901(09)60005-4 20633453

[20] Kreiner M, Zaffaroni A, Alvarez R, Clark G. Validation of a simplified sham acupuncture technique for its use in clinical research: a randomised, single blind, crossover study. Acupuncture in Medicine. 2010;28 (1):33–6. 10.1136/aim.2009.001735 20351375

[21] Tough EA, White AR, Richards SH, Lord B, Campbell JL. Developing and validating a sham acupuncture needle. Acupuncture in Medicine. 2009;27(3):118–22. 10.1136/aim.2009.000737 19734382

[22] Higgins JPT, Green S. Cochrane Handbook for Systematic Reviews of Interventions Version 5.1.0 [updated March 2011]: The Cochrane Collaboration; 2011 Available: http://www.cochrane-handbook.org/.

[23] Bang H, Ni L, Davis CE. Assessment of blinding in clinical trials. Controlled clinical trials. 2004;25(2):143–56. Epub 2004/03/17. 10.1016/j.cct.2003.10.016 . 15020033

[24] Andersen D, Lossl K, Nyboe Andersen A, Furbringer J, Bach H, Simonsen J, et al Acupuncture on the day of embryo transfer: a randomized controlled trial of 635 patients. Reproductive biomedicine online. 2010;21(3):366–72. Epub 2010/07/20. 10.1016/j.rbmo.2010.03.029 . 20638338

[25] Chen L, Mao J, Fernandes S, Galantino M, Guo W, LaRiccia P, et al Integrating acupuncture with exercise-based physical therapy for knee osteoarthritis: a randomized controlled trial. Journal of clinical rheumatology. 2013;19(6):308–16. 10.1097/RHU.0b013e3182a21848 23965480 PMC3782092

[26] Elden H, Fagevik-Olsen M, Ostgaard HC, Stener-Victorin E, Hagberg H. Acupuncture as an adjunct to standard treatment for pelvic girdle pain in pregnant women: randomised double-blinded controlled trial comparing acupuncture with non-penetrating sham acupuncture. BJOG: an international journal of obstetrics and gynaecology. 2008;115(13):1655–68. Epub 2008/10/25. 10.1111/j.1471-0528.2008.01904.x . 18947338

[27] Goldman RH, Stason WB, Park SK, Kim R, Schnyer RN, Davis RB, et al Acupuncture for treatment of persistent arm pain due to repetitive use: a randomized controlled clinical trial. The Clinical journal of pain. 2008;24(3):211–8. Epub 2008/02/22. 10.1097/AJP.0b013e31815ec20f . 18287826

[28] Karst M, Rollnik JD, Fink M, Reinhard M, Piepenbrock S. Pressure pain threshold and needle acupuncture in chronic tension-type headache—a double-blind placebo-controlled study. Pain. 2000;88(2):199–203. Epub 2000/10/26. . 11050375 10.1016/S0304-3959(00)00315-8

[29] Kleinhenz J, Streitberger K, Windeler J, Gussbacher A, Mavridis G, Martin E. Randomised clinical trial comparing the effects of acupuncture and a newly designed placebo needle in rotator cuff tendinitis. Pain. 1999;83(2):235–41. Epub 1999/10/27. . 10534595 10.1016/s0304-3959(99)00107-4

[30] Lathia AT, Jung SM, Chen LX. Efficacy of acupuncture as a treatment for chronic shoulder pain. Journal of alternative and complementary medicine. 2009;15(6):613–8. Epub 2009/06/06. 10.1089/acm.2008.0272 . 19489707

[31] Linde M, Fjell A, Carlsson J, Dahlof C. Role of the needling per se in acupuncture as prophylaxis for menstrually related migraine: a randomized placebo-controlled study. Cephalalgia: an international journal of headache. 2005;25(1):41–7. Epub 2004/12/21. 10.1111/j.1468-2982.2004.00803.x . 15606569

[32] Pariente J, White P, Frackowiak RS, Lewith G. Expectancy and belief modulate the neuronal substrates of pain treated by acupuncture. NeuroImage. 2005;25(4):1161–7. Epub 2005/04/27. 10.1016/j.neuroimage.2005.01.016 . 15850733

[33] Rebhorn C, Breimhorst M, Buniatyan D, Vogel C, Birklein F, Eberle T. The efficacy of acupuncture in human pain models: a randomized, controlled, double-blinded study. Pain. 2012;153(9):1852–62. Epub 2012/06/29. 10.1016/j.pain.2012.05.026 . 22738796

[34] Schliessbach J, van der Klift E, Arendt-Nielsen L, Curatolo M, Streitberger K. The effect of brief electrical and manual acupuncture stimulation on mechanical experimental pain. Pain medicine (Malden, Mass). 2011;12(2):268–75. Epub 2011/02/01. 10.1111/j.1526-4637.2010.01051.x . 21276188

[35] Schliessbach J, Van Der Klift E, Siegenthaler A, Arendt-Nielsen L, Curatolo M, Streitberger K. Does acupuncture needling induce analgesic effects comparable to diffuse noxious inhibitory controls? Evid-Based Complement Altern Med. 2012;2012(785613). . 10.1155/2012/785613 PMC3132481 21760827

[36] Streitberger K, Friedrich-Rust M, Bardenheuer H, Unnebrink K, Windeler J, Goldschmidt H, et al Effect of acupuncture compared with placebo-acupuncture at P6 as additional antiemetic prophylaxis in high-dose chemotherapy and autologous peripheral blood stem cell transplantation: a randomized controlled single-blind trial. Clinical cancer research. 2003;9(7):2538–44. Epub 2003/07/12. . 12855628

[37] Sunay D, Ozdiken M, Arslan H, Seven A, Aral Y. The effect of acupuncture on postmenopausal symptoms and reproductive hormones: a sham controlled clinical trial. Acupuncture in Medicine. 2011;29(1):27–31. Epub 2011/03/09. 10.1136/aim.2010.003285 . 21383392

[38] Sunay D, Sunay M, Aydogmus Y, Bagbanci S, Arslan H, Karabulut A, et al Acupuncture versus paroxetine for the treatment of premature ejaculation: a randomized, placebo-controlled clinical trial. European urology. 2011;59(5):765–71. Epub 2011/01/25. 10.1016/j.eururo.2011.01.019 . 21256670

[39] White P, Bishop FL, Prescott P, Scott C, Little P, Lewith G. Practice, practitioner, or placebo? A multifactorial, mixed-methods randomized controlled trial of acupuncture. Pain. 2011;153(2):455–62. Epub 2011/12/16. 10.1016/j.pain.2011.11.007 . 22169359

[40] White P, Lewith G, Hopwood V, Prescott P. The placebo needle, is it a valid and convincing placebo for use in acupuncture trials? A randomised, single-blind, cross-over pilot trial. Pain. 2003;106(3):401–9. Epub 2003/12/09. . 14659523 10.1016/j.pain.2003.08.013

[41] Yao E, Gerritz PK, Henricson E, Abresch T, Kim J, Han J, et al Randomized controlled trial comparing acupuncture with placebo acupuncture for the treatment of carpal tunnel syndrome. PM & R: the journal of injury, function, and rehabilitation. 2012;4(5):367–73. Epub 2012/03/13. 10.1016/j.pmrj.2012.01.008 . 22405683

[42] Zhang M, Huang G, Lu F, Paulus W, Sterzik K. Effect of acupuncture on the pregnancy rate in embryo transfer and mechanisms: A randomized and control study [in Chinese]. Chinese Acupuncture and Moxibustion. 2003;23(1):3–5.

[43] Zhang W. Clinical Study on Acupuncture at Back-shu Points for Chronic Fatigue Syndrome: A Report of 22 Cases [in Chinese]. Journal of Traditional Chinese Medicine. 2010;51(2):139–41.

[44] Zheng S, Zheng S, Jiao J, Ren R, Wei L, Yang L, et al The effects of acupuncture therapy on back-shu and front-mu points on the quality-of-life of patients with chronic fatigue syndrome [in Chinese]. Guiding Journal of Traditional Chinese Medicine and Pharmacy 2011;17(7):66–8.

[45] Chae Y, Lee H, Kim H, Kim CH, Chang DI, Kim KM, et al Parsing brain activity associated with acupuncture treatment in Parkinson's diseases. Movement disorders. 2009;24(12):1794–802. Epub 2009/06/18. 10.1002/mds.22673 . 19533753

[46] Chou LW, Hsieh YL, Kao MJ, Hong CZ. Remote influences of acupuncture on the pain intensity and the amplitude changes of endplate noise in the myofascial trigger point of the upper trapezius muscle. Archives of physical medicine and rehabilitation. 2009;90(6):905–12. Epub 2009/06/02. 10.1016/j.apmr.2008.12.020 . 19480864

[47] Downs NM, Kirk K, MacSween A. The effect of real and sham acupuncture on thermal sensation and thermal pain thresholds. Archives of physical medicine and rehabilitation. 2005;86(6):1252–7. Epub 2005/06/15. 10.1016/j.apmr.2004.10.037 . 15954068

[48] Kennedy S, Baxter GD, Kerr DP, Bradbury I, Park J, McDonough SM. Acupuncture for acute non-specific low back pain: a pilot randomised non-penetrating sham controlled trial. Complement Ther Med. 2008;16(3):139–46. Epub 2008/06/07. 10.1016/j.ctim.2007.03.001 . 18534326

[49] Modlock J, Nielsen BB, Uldbjerg N. Acupuncture for the induction of labour: a double-blind randomised controlled study. BJOG. 2010;117(10):1255–61. Epub 2010/06/25. 10.1111/j.1471-0528.2010.02647.x ; PubMed Central PMCID: PMCPmc2955967. 20573151 PMC2955967

[50] Rogha M, Rezvani M, Khodami AR. The effects of acupuncture on the inner ear originated tinnitus. Journal of Research in Medical Sciences. 2011;16(9):1217–23. . 22973392 PMC3430048

[51] Smith MJ, Tong HC. Manual acupuncture for analgesia during electromyography: a pilot study. Archives of physical medicine and rehabilitation. 2005;86(9):1741–4. Epub 2005/09/27. 10.1016/j.apmr.2004.11.044 . 16181936

[52] Smith P, Mosscrop D, Davies S, Sloan P, Al-Ani Z. The efficacy of acupuncture in the treatment of temporomandibular joint myofascial pain: a randomised controlled trial. Journal of dentistry. 2007;35(3):259–67. Epub 2006/11/11. 10.1016/j.jdent.2006.09.004 . 17095133

[53] So EW, Ng EH, Wong YY, Yeung WS, Ho PC. Acupuncture for frozen-thawed embryo transfer cycles: a double-blind randomized controlled trial. Reproductive biomedicine online. 2010;20(6):814–21. Epub 2010/04/13. 10.1016/j.rbmo.2010.02.024 . 20382081

[54] So EWS, Ng EHY, Wong YY, Lau EYL, Yeung WSB, Ho PC, et al A randomized double blind comparison of real and placebo acupuncture in IVF treatment. Deutsche Zeitschrift fur Akupunktur. 2009;52(1):49–50. . 10.1093/humrep/den380 18940896

[55] Suzuki M, Muro S, Ando Y, Omori T, Shiota T, Endo K, et al A randomized, placebo-controlled trial of acupuncture in patients with chronic obstructive pulmonary disease (COPD): the COPD-acupuncture trial (CAT). Archives of internal medicine. 2012;172(11):878–86. Epub 2012/08/21. 10.1001/archinternmed.2012.1233 . 22905352

[56] Tong J, Chen J, Zhang Z, Pan Y, Zheng J, Yao H. The evaluation of the clinical effect of acupuncture treatment of simple obesity [in Chinese]. Journal of Guangzhou University of Traditional Chinese Medicine. 2010;27(6):579–82.

[57] Tong J, Yao H, Chen J, Zhang Z, Huang S. Clinical Observation on the Therapeutic Effect of Abdominal Acupuncture Therapy for Simple Obesity [in Chinese]. Acupuncture Research. 2006;31(3):176–78.

[58] Takakura N, Yajima H. Analgesic effect of acupuncture needle penetration: a double-blind crossover study. Open medicine. 2009;3(2):e54–61. 19946394 PMC2765771

[59] Vase L, Baram S, Takakura N, Yajima H, Takayama M, Kaptchuk TJ, et al Specifying the nonspecific components of acupuncture analgesia. Pain. 2013;154(9):1659–67. Epub 2013/05/28. 10.1016/j.pain.2013.05.008 ; PubMed Central PMCID: PMCPmc3981538. 23707680 PMC3981538

[60] Bang H, Flaherty SP, Kolahi J, Park J. Blinding assessment in clinical trials: A review of statistical methods and a proposal of blinding assessment protocol. Clin Res Regul Aff. 2010;27(2):42–51. 10.3109/10601331003777444

[61] Freed B, Assall OP, Panagiotakis G, Bang H, Park JJ, Moroz A, et al Assessing blinding in trials of psychiatric disorders: A meta-analysis based on blinding index. Psychiatry Res. 2014;219(2):241–7. 10.1016/j.psychres.2014.05.023 24930582 PMC4183143

[62] MacPherson H, Vertosick E, Lewith G, Linde K, Sherman KJ, Witt CM, et al Influence of control group on effect size in trials of acupuncture for chronic pain: a secondary analysis of an individual patient data meta-analysis. PLoS One. 2014 4 4;9(4):e93739 10.1371/journal.pone.0093739 eCollection 2014. 24705624 PMC3976298

[63] Moroz A, Freed B, Tiedemann L, Bang H, Howell M, Park JJ. Blinding measured: a systematic review of randomized controlled trials of acupuncture. Evid Based Complement Alternat Med. 2013;2013:708251 10.1155/2013/708251 Epub 2013 Mar 3. 23533515 PMC3603669

[64] Gong XL, Pan ZH, Shen Y, Wang S. Blinding effect of non-penetrating sham needle in the randomized controlled trials of acupuncture: A systematic review. Journal of Acupuncture and Tuina Science. 2014;12(1):8–11.

[65] Park J, White AR, James MA, Hemsley AG, Johnson P, et al Acupuncture for subacute stroke rehabilitation: a Sham-controlled, subject- and assessor-blind, randomized trial. Arch Intern Med: 2005; 165: 2026–2031. 16186474 10.1001/archinte.165.17.2026

[66] Enblom A, Johnsson A, Hammar M, Steineck G, Borjeson S. The nonpenetrating telescopic sham needle may blind patients with different characteristics and experiences when treated by several therapists. Evidence-based Complementary and Alternative Medicine: 2011. 10.1155/2011/185034 PMC3124016 21747890

[67] Enblom A, Hammar M, Steineck G, Borjeson S. Can individuals identify if needling was performed with an acupuncture needle or a non-penetrating sham needle? Complement Ther Med: 2008; 16: 288–294. 19186344 10.1016/j.ctim.2008.02.012

[68] Takakura N, Yajima H. A placebo acupuncture needle with potential for double blinding—a validation study. Acupuncture in Medicine. 2008;26(4):224–30. Epub 2008/12/23. . 19098693 10.1136/aim.26.4.224

[69] Takakura N, Takayama M, Kawase A, Kaptchuk TJ, Yajima H. Double blinding with a new placebo needle: a further validation study. Acupuncture in Medicine. 2010;28(3):144–8. Epub 2010/06/10. 10.1136/aim.2009.001230 ; PubMed Central PMCID: PMCPmc2933308. 20530096 PMC2933308

[70] Takakura N, Takayama M, Kawase A, Yajima H. Double blinding with a new placebo needle: a validation study on participant blinding. Acupuncture in Medicine. 2011;29(3):203–7. Epub 2011/03/16. 10.1136/aim.2010.002857 . 21402558

[71] Manni L, Albanesi M, Guaragna M, Barbaro Paparo S, Aloe L. Neurotrophins and acupuncture. Autonomic Neuroscience. 2010;157(1–2):9–17. 10.1016/j.autneu.2010.03.020 20451467

[72] Dorsher PT. Invited commentary on "discrimination of real and sham acupuncture needles using the park sham device: A preliminary study". Archives of physical medicine and rehabilitation. 2010;91(8):1306–8. 10.1016/j.apmr.2010.04.025 20684914

[73] Vase L, Baram S, Takakura N, Takayama M, Yajima H, Kawase A, et al Can acupuncture treatment be double-blinded? An evaluation of double-blind acupuncture treatment of postoperative pain. PLoS One. 2015 3 6;10(3):e0119612 10.1371/journal.pone.0119612 eCollection 2015. 25747157 PMC4352029

[74] Bishop FL, Lewith GT. A review of psychosocial predictors of treatment outcomes: what factors might determine the clinical success of acupuncture for pain? Journal of Acupuncture and Meridian Studies. 2008;1(1):1–12. Epub 2008/09/01. 10.1016/s2005-2901(09)60001-7 . 20633449

