# Supplementary material for: Placebo Devices as Effective Control Methods in Acupuncture Clinical Trials: A Systematic Review
Source: PLoS One. 2015 Nov 4;10(11):e0140825. doi: 10.1371/journal.pone.0140825 (PMC4633221; doi:10.1371/journal.pone.0140825)
Supplement: S1 Table — (DOCX) [file pone.0140825.s002.docx]

**Supplementary file (S1): search strategy**

**Pubmed**

#1. Randomized controlled trial [Publication Type])

#2. Controlled clinical trial [Publication Type])

#3. Clinical trials [MeSH Major Topic])

#4. #2 OR #3

#5. Randomized [Title/Abstract])

#6. Random* [Title/Abstract])

#7. #5 OR #6

#8. #4 AND #7

#9. #1 OR #8

#10. Acupuncture [Title/Abstract])

#11. Acupuncture [MeSH Terms]

#12. Acupuncture therapy [MeSH Terms]

#13. Acupunc* [Title/Abstract])

#14. #10 OR #11 OR #12 OR #13

#15. #9 AND #14
